# Conventional Versus Minimally Invasive Veneers: A Systematic Review

**DOI:** 10.7759/cureus.44638

**Published:** 2023-09-04

**Authors:** Abbasi Begum Meer Rownaq Ali

**Affiliations:** 1 Department of Prosthodontics, Riyadh Elm University, Riyadh, SAU

**Keywords:** minimal-prep veneers, color stability, marginal fit, microleakage, patient satisfaction, esthetic longevity, prosthodontics, no-preparation veneers, conventional veneers, veneers

## Abstract

This systematic review aimed to synthesize and analyze a collection of studies focused on comparing conventional veneers (CVs) and minimal or no-preparation veneers (MPVs) within the domain of prosthodontics. The review sought to explore various attributes, techniques, outcomes, and considerations associated with these two approaches. The key aspects investigated included esthetics, longevity, periodontal health, patient satisfaction, structural influences such as microleakage and marginal fit, cement thickness, and color stability. A systematic literature search was conducted to identify relevant studies published up to the present date. Studies meeting specific inclusion and exclusion criteria were selected for review. Studies pertaining to various methodologies were identified initially as part of the strategy and bias assessment was performed to determine the credibility of their assessments before inclusion in this review. Four comparative assessments gleaned from the selected studies provided a comprehensive overview of the strengths and limitations associated with CVs and MPVs. MPVs demonstrated advantages such as enhanced survival rates and extended mean success periods, implying their potential as viable long-term restorative options. Meticulous patient management and optimal preparation techniques emerged as crucial factors influencing successful outcomes. Structural attributes, including microleakage and marginal fit, varied depending on the preparation techniques employed. Moreover, considerations related to color changes in veneers underscored the intricate interplay between material properties and fabrication methods. The insights underscore the significance of patient-centric care, precision preparation methodologies, and material choices in guiding successful outcomes. However, the diverse methodologies and constraints of certain studies warrant careful interpretation. This study accentuates the potential for future research directions, interdisciplinary collaboration, and the advancement of evidence-based practices within veneer dentistry.

## Introduction and background

Continuous improvements in restorative dentistry have been made in the field of prosthodontics, and veneer treatments have become a crucial component in improving dental function and appearance [1]. Conventional veneers (CVs) have been used for a long time as the go-to method for repairing tooth structures and enhancing dental aesthetics [2]. A paradigm change in restorative procedures has been brought about by the introduction of minimally invasive or no-preparation veneers (MPVs), which hold the promise of preserving natural tooth structure while obtaining equivalent cosmetic results [3]. Modern advancements in ceramic material characteristics and adhesive bonding techniques have significantly accelerated the refining of treatment modalities, enhancing therapeutic efficacy and prospects [1]. MPVs have gained popularity and are distinguished by their thin thickness, which ranges from 0.2 to 0.5 mm [2-4]. CVs, on the other hand, range in thickness from 0.3 to 1.0 mm [5-7], indicating a more substantial construction.

The adoption of ultrathin, or contact lens, veneers, which do not require extensive dental preparation, is a significant modern trend [8]. These veneers offer substantial color integrity and a long-lasting aesthetic impact despite their small sizes [9]. Modern adhesive techniques have strengthened the ability of porcelain substrates to adhere seamlessly, making it easier to finish and shape the gingival peripheries precisely [10]. MPVs have a strong biocompatibility with dental substrates, which coincides with their propensity to collect little bacterial plaque and encourage better oral hygiene [11,12]. The dogma of traditional veneers, on the other hand, calls for extensive dental reduction; however, no-prep and minimally invasive variations circumvent this requirement by requiring just minimum or no enamel ablation. Notably, cases of tooth protrusion, positional alterations, or severe dental crowding may necessitate additional orthodontic treatments [11,13].

MPVs have many benefits, including a significant reduction in discomfort or pain during the procedure [10]. Their clinical appeal is frequently provided without the necessity for anesthesia, easy impression-taking, and eliminated interim restorations. Additionally, by avoiding temporary restorations, patients experience comfort in the maintenance of their natural dental architecture [5,7,11].

Feldspathic porcelain is invariably the chosen material substrate for MPVs, making it possible to create ultrathin veneers with a thickness of 0.2-0.3 mm. As a result of their varied procedure requirements, heavier pressed ceramics measuring 0.3-0.5 mm necessitate a longer journey of dental structure reduction [12]. The landscape of clinical inquiry shows strong opinions of traditional veneers, which is highlighted by the dearth of clinical inquiries into genuine no-prep veneers [13].

Keeping the above in mind, this systematic review aims to define the distinctive characteristics of CVs and MPVs; thoroughly describe the methodologies used in the studies under investigation; synthesize the reported results in terms of esthetics, longevity, periodontal health, patient satisfaction, structural considerations, and color stability; and illuminate the potential drawbacks and restrictions of these techniques. This review attempts to offer a thorough overview that enables a holistic knowledge of the benefits, constraints, and factors influencing the decision between CVs and MPVs by methodically evaluating the body of existing literature.

## Review

Methodology

PRISMA Protocol

**Figure 1:**
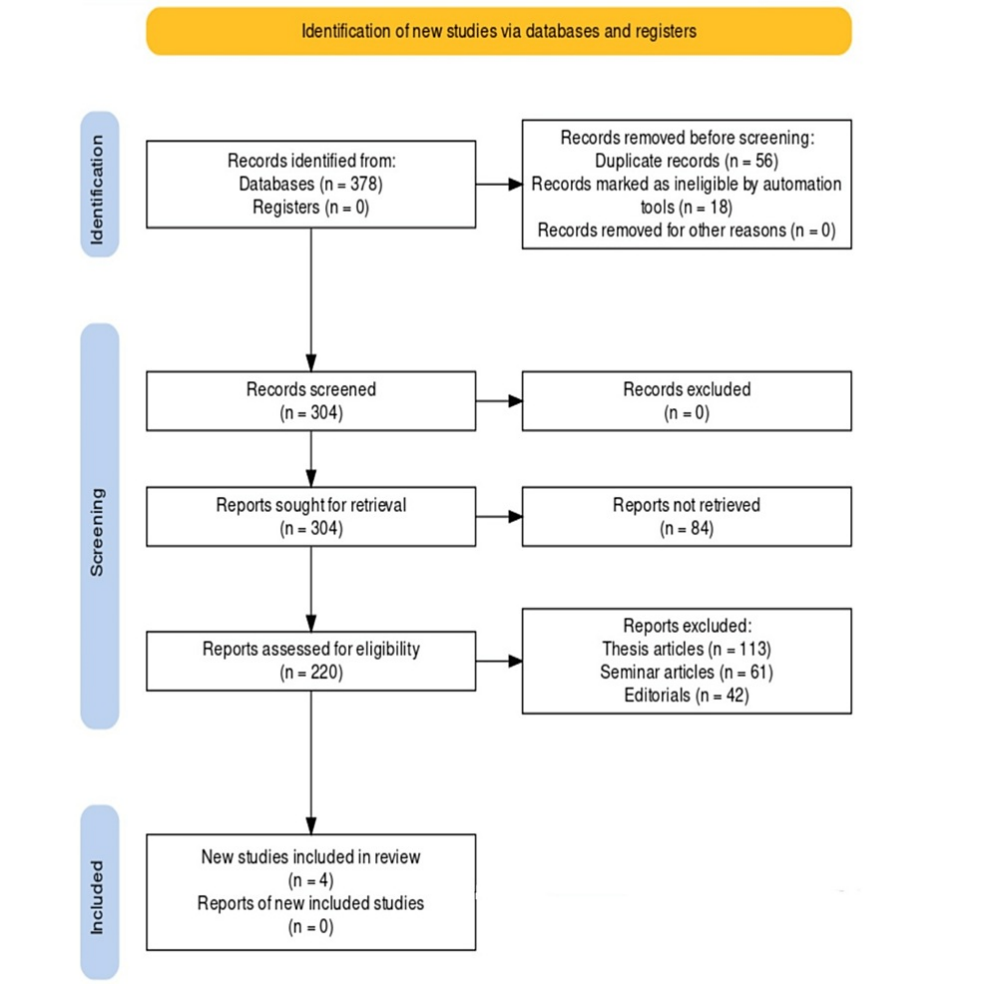
Representation of the study selection process using the Preferred Reporting Items for Systematic Reviews and Meta-Analyses.

This review was performed per the Preferred Reporting Items for Systematic Reviews and Meta-Analyses (PRISMA) standards [14]. The review aimed to thoroughly analyze and summarize the literature that contrasts CVs and MPVs. The PRISMA protocol was painstakingly followed, including a step-by-step procedure to ensure strict methodology and open reporting, as represented in Figure 1. A comprehensive literature search was undertaken to start the review across a number of electronic databases, including PubMed, Scopus, and Web of Science.

PECOS Protocol

For this review, the Population, Exposure, Comparison, Outcome, Study (PECOS) methodology was painstakingly developed to enable a thorough and rigorous study of the research landscape. The inclusion criteria were strict and covered papers that fit certain criteria. Studies in the field of prosthodontics made up the population of interest, with a key emphasis on clinical comparisons between CVs and MPVs. The systematic review sought research discussing the unique clinical effects of these two veneer methods regarding exposure. Additionally, studies that clarified the relative longevity, marginal fit, color stability, patient satisfaction, and problems related to both CVs and MPVs were included in the comparison criteria. The result criteria covered research projects that reported at least a one-year follow-up period, guaranteeing that longitudinal observations were crucial in the evaluation of the aforementioned clinical outcomes. The physical characteristics and bonding capabilities of both CVs and MPVs were examined in vitro investigations to broaden the scope and contribute to a thorough understanding of the underlying mechanisms influencing clinical outcomes.

Hypothesis/Research Question

CVs had better clinical outcomes than MPVs.

Search Strategy

Eight databases were thoroughly searched for this systematic review. Boolean operators and Medical Subject Headings (MeSH) keywords were strategically combined in the search strategy to extract all pertinent studies for the review. PubMed, Embase, Scopus, Web of Science, Cochrane Library, CINAHL, ProQuest, and Google Scholar were among the databases used. The search criteria were set up to include studies directly comparing MPVs with CVs in the prosthodontics field. As shown in Table 1, to increase search accuracy and inclusiveness, several search phrases and concepts were combined using the Boolean operators “AND” and “OR.” In the search approach, the following MeSH terms and keywords were used: “veneers,” “conventional veneers,” “minimal prep veneers,” “no-preparation veneers,” “prosthodontics,” “aesthetic longevity,” “microleakage,” “marginal fit,” “patient satisfaction,” “colour stability,” and “clinical outcomes.”

**Table 1:** The search protocol across different databases.

Database	Search string
PubMed	(“veneers”[MeSH Terms] OR “veneers”[All Fields]) AND (“conventional veneers”[All Fields] OR “minimal prep veneers”[All Fields] OR “no-preparation veneers”[All Fields]) AND (“prosthodontics”[MeSH Terms] OR “prosthodontics”[All Fields])
Embase	(‘veneers’/exp OR ‘veneers’) AND (‘conventional veneers’ OR ‘minimal prep veneers’ OR ‘no-preparation veneers’) AND (‘prosthodontics’/exp OR ‘prosthodontics’)
Scopus	(TITLE-ABS-KEY (“veneers”) AND TITLE-ABS-KEY (“conventional veneers” OR “minimal prep veneers” OR “no-preparation veneers”) AND TITLE-ABS-KEY (“prosthodontics”))
Web of Science	TS=(“veneers”) AND TS=(“conventional veneers” OR “minimal prep veneers” OR “no-preparation veneers”) AND TS=(“prosthodontics”)
Cochrane Library	“veneers” AND (“conventional veneers” OR “minimal prep veneers” OR “no-preparation veneers”) AND “prosthodontics”
CINAHL	(MH “veneers”) AND (“conventional veneers” OR “minimal prep veneers” OR “no-preparation veneers”) AND (MH “prosthodontics”)
ProQuest	AB(veneers) AND AB(“conventional veneers” OR “minimal prep veneers” OR “no-preparation veneers”) AND AB(prosthodontics)
Google Scholar	intitle:veneers AND (“conventional veneers” OR “minimal prep veneers” OR “no-preparation veneers”) AND intitle:prosthodontics

Inclusion and Exclusion Criteria

Studies that offered insights into a wide range of traits were essential to the criteria. These qualities included durability, a critical factor in determining the prolonged efficacy of veneers, and a crucial aspect of marginal fit, which determined the accuracy and stability of the dentition after veneer restoration. Additionally, the criteria were effectively broadened to include color stability, a factor crucial to the aesthetic dimension. Within the context of these criteria, patient satisfaction, a crucial indicator of the quality of the patient experience and the effectiveness of the treatment, took center stage. The inclusion criteria also recognized the importance of addressing issues that might conceivably occur during the installation of veneers, providing a thorough assessment of the intervention’s effects. To ensure the accuracy and integrity of the review, the exclusion criteria were defined concurrently with the inclusion criteria. Notably, studies that did not specifically compare MPVs with CVs were routinely disregarded. The exclusion criteria also required that research be presented in the English language, which was in line with the intellectual environment of the review and guaranteed a coherent assimilation of findings.

Data Extraction Protocol

The main aim of this protocol was to thoroughly extract pertinent information about the comparison of CVs and MPVs from the chosen studies. This included attributes, methods, interventions, key findings, and other relevant data. The construction of a thorough data extraction form with predefined fields for the extraction of information served as the foundation for the data extraction methodology. These areas were created to record important information such as study goals, sample size, design, patient characteristics, interventions, measured outcomes, findings, and conclusions. To ensure that the form was complete and suitable for data collection, a subset of studies served as the basis for a full pilot test. An interrater reliability test was performed to increase the accuracy of the data extraction procedure. The test was administered by two impartial assessors with backgrounds in prosthodontics and dental research. The predefined data extraction form was used by both reviewers to independently extract data from a preset group of studies that were chosen at random. Cohen’s Kappa was used to measure the inter-rater reliability, and a result of 0.85 indicated that there was significant agreement between the reviewers. Any differences or disputes in data extraction were reviewed and settled by consensus between the two reviewers once the interrater reliability test was complete. The goal of this iterative process was to improve the data extraction protocol and ensure accuracy and uniformity in the extraction procedure. The primary data extraction procedure was then completed independently by the reviewers using the approved data extraction form. The retrieved data were carefully cross-checked to find any inconsistencies and fix them. When questions emerged, the reviewers discussed it until they came to an agreement.

Bias Evaluation Protocol

**Figure 2:**
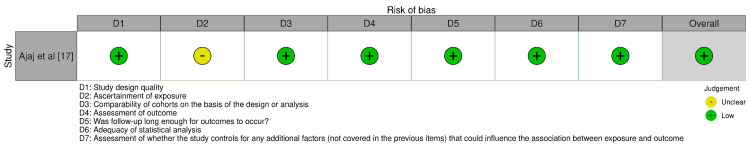
Assessment of bias for the case series included in the review.

**Figure 3:**
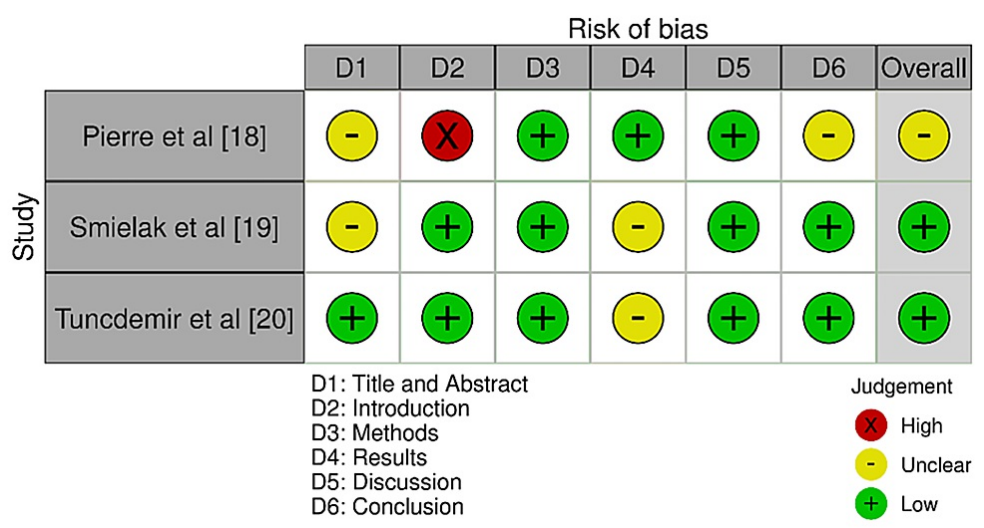
Assessment of bias for the in-vitro papers included in the review.

In this review, clinical studies were assessed using the Newcastle-Ottawa Scale (NOS) [15], while in-vitro research was reviewed using a modified version of the Consolidated Standards of Reporting Trials (CONSORT) tool [16]. Both of these tools and their respective bias assessment domains are represented in Figure 2 and Figure 3, respectively.

Results

In the initial stage, potential studies were thoroughly identified from databases and registries. This search produced a large collection, with a total of 378 records coming from databases but none from registers. Due to the strictness of this initial screening technique, 56 duplicate entries had to be removed, and 18 records had to be removed because automated programs had marked them as being ineligible. In parallel, an excursion into uncharted territory produced 12 records from websites and 27 records from citation searches. The subsequent phase led to the screening of the cumulative corpus, which included 304 documents, and involved a rigorous examination to guarantee alignment with the review’s goals. None of the records were rejected for inclusion and were later kept for closer examination. This led to the careful retrieval of 304 reports. The research selection procedure moved to the eligibility assessment stage, which was marked by a rigorous evaluation of 220 papers. The strict eligibility requirements were put to the test during this crucial stage, which ultimately shaped the landscape of inclusion and exclusion. A total of 113 reports were excluded because they were classified as thesis pieces, 61 reports as seminar articles, and 42 reports as editorials. Following this evaluation, 27 were included to proceed to the last stage of the research selection process. Nineteen papers were eliminated because they were categorized as literature reviews, and eight papers were excluded as they were inconsistent with the stated goals. Four papers [17-20] were finally accepted and included in the review.

The demographic details of the included studies [17-20] are presented in Table 2. The study included a small sample size of four entities, utilized a case series approach, and methodically covered a follow-up period of three years, allowing the identification of long-term patterns, starting with the examination done by Ajaj [17]. Pierre et al. [18] conducted an in-vitro study in 2023. Although the study’s sample size was increased to 24, the follow-up period was not specified, which would have limited the scope of the study. Smielak et al. [19] started a project in 2022 with 186 patients for an in-vitro examination. The scientific study conducted in the year 2020 by Tuncdemir et al. [20] included a sample of 40 individuals.

**Table 2:** Demographic characteristics of the included papers.

Study	Year	Sample size (n)	Protocol	Follow-up period
Ajaj [17]	2020	4	Case series	Five years (mean)
Pierre et al. [18]	2023	24	In vitro	Unspecified
Smielak et al. [19]	2022	186	In vitro	Nine years
Tuncdemir et al. [20]	2020	40	In vitro	Unspecified

A thorough knowledge of the complex dynamics within the field of veneer dentistry is made possible by the combined assessments resulting from the comparative analyses of the studies [17-20], as shown in Table 3. These evaluations emphasize the importance of carefully planning patient management techniques to achieve positive results. The common theme among the studies emphasizes that MPVs show significant advantages over CVs, particularly in terms of survival rates and lifetime [17]. The significantly higher survival rates and mean success rates shown in the MPV groups support the idea that less preparation can result in better clinical outcomes [18]. As shown by the difference in cement thickness among groups with various preparation approaches, the assessments together emphasize the critical role of preparation techniques in determining the structural characteristics of veneers. The research on microleakage and marginal fit supports the significance of exact preparation techniques and their effect on the general integrity of veneer restorations [19]. To minimize potential variations over time, careful material selection and construction techniques are essential. The analyses also highlight the complexities surrounding color stability and changes in veneers [20].

**Table 3:** Comparison between the different parameters of traditional and no-prep veneers observed in the selected studies.

Study	Objective	Methods	Interventions	Key findings	Conclusions
Ajaj [17]	To compare traditional veneers and no-prep veneers in terms of attributes, steps, and outcomes	Analyzed four no-prep veneer cases made of Cerinate feldspathic pressable porcelain. Followed patients for seven years, and assessed esthetic longevity, periodontal health, patient satisfaction, and treatment impact	No-prep veneers (Cerinate)	- Meticulous oral hygiene and follow-up are key for periodontal health	No-prep veneers had advantages but required careful patient management
				- Cleaning gingival and interproximal areas is challenging	
				- Fracture/chipping risks at thin gingival margins	
				- Adequate adaptation is crucial for success	
				- Treatment is highly satisfactory with avoidable adverse outcomes	
Pierre et al. [18]	To evaluate the influence of preparation techniques on microleakage, marginal fit, and cement thickness of lithium disilicate veneers	Divided 24 maxillary central incisors into groups with minimally invasive preparation and no preparation. Restored with veneers and assessed microleakage, marginal fit, and cement thickness after aging	Minimally invasive preparation	- Significant microleakage at the cervical vs. proximal area	The preparation technique affected cement thickness but not microleakage or marginal fit
				- Similar marginal fit in both groups	
				- Reduced cement thickness with minimally invasive prep at the cervical area	
Smielak et al. [19]	To compare the survival rates of conventional vs. no-prep/minimally invasive porcelain veneers	Placed 186 veneers (conventional and no-prep/minimally invasive) in 35 patients. Evaluated restorations over nine years	Conventional veneers (84) and no-prep/minimally invasive veneers (102)	- Higher survival rate (100%) for no-prep/minimally invasive veneers vs. conventional veneers (9.67%)	No-prep/minimal-prep veneers showed higher survival rates over nine years compared to conventional veneers
				- Absolute failures: chipping/fractures, debonding, and tooth fracture	
				- Longer mean success rate for no-prep veneers (10.28 vs. 9.32 years)	
Tuncdemir et al. [20]	To determine the effects of preparation vs. non-preparation and porcelain type on color changes in laminate veneers	Used 40 maxillary incisors in four groups (preparation/non-preparation with different porcelain types). Measured initial and post-accelerated aging color changes	IPS e.max CAD prep	- Preparation caused more color changes than nonpreparation	Preparation caused more color changes in veneers, and certain fabrication methods increased color change after aging
				- IPS e.max CAD for nonprepared PLVs increased color change after aging	

The overall conclusions drawn from the studies listed in the table provide crucial insights into the field of veneer dentistry and have an impact on clinical practice, material choice, and treatment procedures. Ajaj [17] highlighted the critical importance of diligent patient management and dental hygiene in achieving effective outcomes in no-prep veneer cases by contrasting CVs with MPVs. To reduce the risk of chipping, fracture, and gingival irritation associated with this method, careful patient education and follow-up sessions are essential. These results are significant because they highlight the imperative need for sufficient adaptation to guarantee the clinical viability of no-prep veneers. Pierre et al. [18] investigated how preparation methods affect cement thickness, marginal fit, and microleakage to understand how veneer restorations are structurally supported. The study underlined the significance of accurate preparation procedures in maintaining the integrity of the veneer and minimizing potential issues by revealing considerable microleakage in the cervical area and lower cement thickness with minimally invasive preparation. These results have ramifications for clinical procedures and can help improve preparatory methods for the best veneer results. The survival rates of CVs and MPVs were compared by Smielak et al. [19], who clarified the lifetime benefits of minimal preparation techniques. The astounding 100% survival rate and extended mean success rate seen in the MPV group support the potential clinical advantages of using these methods. These findings support the development of evidence-based guidelines and foster positive outcomes and long-term patient satisfaction in addition to informing treatment choices. The investigation into color variations in veneers by Tuncdemir et al. [20] adds to the aesthetic factors supporting veneer dentistry. The differences in color shifts between preparation and non-preparation procedures, which are made worse by manufacturing methods, highlight the difficulties associated with color stability.

The study by Ajaj [17] aimed to contrast the characteristics, methods, and results of standard veneers versus no-prep veneers. The study tracked four cases over a seven-year period using Cerinate feldspathic pressable porcelain for the latter, assessing aesthetics, longevity, periodontal health, patient satisfaction, and therapeutic impact. The results clarified key success criteria, highlighting strict dental cleanliness, ideal adaptation, and vigilance in handling the inherent hazards of chipping and fracture linked with the extremely thin gingival edge of no-prep veneers. These results highlighted the need for thorough patient management to produce positive outcomes. Pierre et al. [18] examined how different preparation methods affected the cement thickness, marginal fit, and microleakage in lithium disilicate veneers. The study investigated the effects of the interventions on these parameters by dividing 24 maxillary central incisors into groups with minimally invasive preparation and without preparation. Significant microleakage was seen in the cervical area relative to the proximal area, which highlights the possible clinical implications of this occurrence. Additionally, the examination at the cervical region showed lower cement thickness with minimally invasive preparation, illuminating the interaction between preparation methods and veneer structure. These results demonstrated the significance of exact veneer preparation and cementation methods in shaping veneer results. Smielak et al. [19] compared the nine-year survival rates of conventional and no-prep/minimally invasive porcelain veneers. The study found that the no-prep/minimal-prep veneers had a noticeably greater survival rate than traditional veneers, with 186 veneers applied to 35 patients. Absolute failures in the traditional veneer group, including chipping, fractures, debonding, and tooth fractures, served as the foundation for this conclusion. The fact that no-prep veneers had a significantly higher mean success rate than traditional veneers attests to the latter’s superior long-term results. These findings highlighted the advantages and clinical practicality of using minimal preparation approaches to extend the life of veneer restorations. Tuncdemir et al. [20] aimed to determine how preparation techniques and porcelain types affected color changes in laminate veneers. They used 40 maxillary incisors and 40 different porcelain varieties separated into four groups based on preparation and non-preparation. The analysis of the initial and post-accelerated aging color changes was part of the experiment. The results specifically showed that preparation caused more color changes than non-preparation.

Discussion

The field of prosthodontics has made constant advancements in restorative dentistry, and veneer treatments have emerged as a key strategy for enhancing dental function and aesthetics. CVs began to show signs of complexity, including an interaction between ceramic chipping, crowning fracturations, and the debonding phenomena that cause dislodgment. It is important to recognize that the annals of clinical engagement contain a unique occurrence when a single patient struggled with four shattered veneers that were the result of a traumatic experience [21]. In addition, a noteworthy interlude that caused the loss of two veneers as a result of the acquisition of X-ray images prompted a possible connection [22]. Importantly, the course of this incident impacts areas of dental topography outside the scope of veneers, going beyond their coverage ambit [23].

The spectrum of failure, especially in the crucible of trauma-ordained setbacks, merits nuanced apprehension in light of this narrative as it is a multifaceted tableau where sole attributions to reduced dentin adhesion remain problematic [24]. Emerging knowledge reveals that the contours of the outcome can be permanently shaped by the veneer’s interaction with a dentin substrate equipped with increased elasticity [25]. The joining of a veneer with dentin that has increased pliability under the gravitational pull of loading may result in increased stress concentrations, creating an ideal environment for the emergence of fracture propensities [26]. Veneers, on the other hand, are positioned to navigate the contours of load-induced stress with more resilience and buoyancy when they are entrenched upon the bedrock of enamel’s unyielding embrace [27-29].

While the research listed in the review gives useful information for veneer dentistry, they are not without limitations that should be taken into account. While offering in-depth observations of no-prep veneer cases, the study by Ajaj [17] was limited by its small sample size of only four cases. Because of the potential effects of the small cohort size, extending the results to larger groups requires careful interpretation. Furthermore, the use of the retrospective case series approach raises the possibility of bias and confounding factors, which can affect the presented results. While favorable in controlled conditions, the in-vitro experiment by Pierre et al. [18] may not accurately duplicate the complicated oral environment. Utilizing removed teeth for laboratory testing may result in variations in material behavior and bonding properties from real-world clinical settings. The study’s vague follow-up duration further restricts our ability to comprehend the long-term effects of preparation methods on veneer outcomes. Despite having a sizable sample size, the study by Smielak et al. [19] was a retrospective analysis relying on pre-existing clinical data. The stated survival rates and outcomes may be impacted by potential issues with data completeness, follow-up consistency, and selection bias introduced by the retrospective approach. Additionally, the variation in patient profiles and features within the sample may be a confounding factor affecting the results. An in-vitro setting was used by Tuncdemir et al. [20] to investigate color changes in veneers. This method may not accurately recreate the oral environment and the many interactions that occur in the mouth, despite being helpful for controlled studies. The inability to determine the durability of color changes over long durations is due to the unclear follow-up period. The complexity of intraoral dynamics is also intrinsically absent from the in-vitro approach, which may limit the applicability of the findings in clinical contexts.

The knowledge of veneer aesthetics guides dentists toward achieving the best results through material choice, production techniques, and treatment planning. Additionally, the overall conclusions drawn from these investigations support the need for interdisciplinary investigations. The creation of holistic treatment modalities that go beyond accepted boundaries is promised by the fusion of prosthodontic, periodontic, and cosmetic dentistry. The opportunity for longitudinal monitoring in the field of veneer outcomes emerges as the research environment continues to change. Extending follow-up times could give practitioners and patients priceless information about the long-term durability and success of veneer therapies.

## Conclusions

Overall, the detailed examination of the studies included in this review enables a diverse comprehension of numerous facets pertaining to veneer dentistry. The findings, which encompass qualities, methods, durability, structural influences, and aesthetic concerns, collectively shed light on crucial ideas that have significant repercussions for clinical practice as well as research. The results underline the importance of carefully managing patients, using the best preparatory methods, and choosing the right materials for veneer procedures. The benefits of no-prep and minimal-prep veneers, as shown by better survival rates and extended mean success rates, support the efficacy of these methods in lengthening the lifespan of veneer restorations. Additionally, the careful examination of color variations within veneers deepens our understanding of aesthetic factors that influence material selection and fabrication methods. Collectively, these findings help shape treatment regimens and patient care methods through evidence-based decision-making. The knowledge gained from these studies ultimately aids in the field’s continued development and innovation, ensuring that veneer restorations continue to be at the forefront of modern prosthodontic practice.
